# Virtual currencies: different schemes and research opportunities

**DOI:** 10.1007/s11002-022-09620-z

**Published:** 2022-02-14

**Authors:** Gianluca Scheidegger, Priya Raghubir

**Affiliations:** 1 Institute of Retail Management, University of St. Gallen, St. Gallen, Switzerland; 2 Leonard N. Stern School of Business, New York University, New York City, NY USA

**Keywords:** Virtual currencies, Pricing, Consumer behavior

## Abstract

The digitization of money has led to the emergence of numerous virtual currencies. Despite their great financial relevance, virtual currencies have not received much attention in marketing research. We classify virtual currencies into three different schemes and highlight potential factors that influence consumer behavior related to these new payment methods. This article provides marketing researchers with a research framework as well as specific research questions to initiate discussions and future research on this novel topic.

## Introduction

Whenever people think about money, their home country’s official currency, real economic money (REM), typically comes to mind first. Yet, many consumers generate non-cash resources, and businesses generate value through points, miles, and other virtual currencies (VCs) that form the backbone of their loyalty programs. These VCs encourage consumer engagement, and offer consumers real goods and services, as well as social standing and the perks accompanying them.

**Fig. 1:**
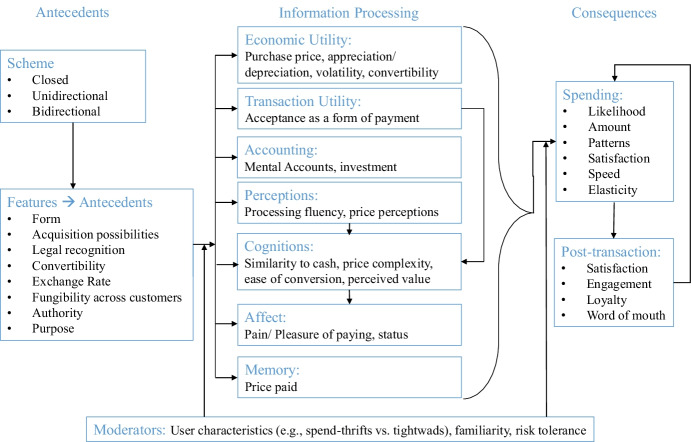
Framework to guide future research on virtual currencies

Gaming, an industry forecast to be worth $314.40 billion by 2027^1^ commonly uses VCs (e.g., CandyCrush gold bars). Then, there are VCs such as Pearls at the music festival Tomorrowland in Belgium, retailer store credits (e.g., BestBuy) and crypto currencies (e.g., bitcoin) that have started being advertised, despite concerns.^2^ Given their financial relevance, VCs remain understudied. We introduce different VC schemes that vary across eight dimensions (Table 1), and suggest areas for research across seven information-processing stages (Fig. 1), with consequences for spending and post-purchase attitudes and behaviors. Payment systems (e.g., credit cards, Venmo) are out of the scope of this article.

**Table 1:** Virtual currency schemes

	Cash (real economic money or REM)	Virtual currency (VC)								
		Closed system	Unidirectional flow					Bidirectional flow		
Short description	Physical money (also available in digital form) that is considered legal tender in an economy	VC (virtual currency) with no connection to REM (real economic money)	A VC (virtual currency) that has a direct (buying VC with REM) or indirect connection to REM (earning VCs through spending REM). VC cannot be converted back to REM (real economic money)					A VC (virtual currency) that can both be bought with, and converted back to REM (real economic money)		
Form	Physical and digital	Digital (Virtual)	Digital (Virtual)					Digital (Virtual)		
Acquisition possibilities	Earned (e.g., Salary, Wages, tips, investment returns, profit) and unearned (e.g., windfall gains, lottery, inheritance)	Engagement	Indirect and direct purchase			Direct purchase and engagement	Indirect purchase	Direct purchase		
Legal recognition	Legal tender	Unrecognized	Unrecognized					Unrecognized		Mostly unrecognized
Convertibility**	Yes	No	Limited	No				Yes		
REM Exchange rate	-	-	-					Floating	Pegged	Floating
Fungibility across customers***	Yes	It depends	Fees may apply			No		Yes		
Authority	Central bank	Issuing company	Issuing company					Issuing company		Decentralized
Purpose	Buy virtual and physical goods and services, store and invest, exchange	Buy virtual goods and services within the system of the issuing company	Buy virtual and physical goods and services within the system of the issuing company and its partners and improve loyalty status		Buy virtual goods and services within the system of the issuing company		Buy virtual and physical goods and services within the system of the issuing company	Buy virtual goods and services within the system of the issuing company	Buy physical goods and services within the system of the issuing company	Buy virtual and physical goods and services outside the system of the issuing authority
Example	Real economy money	Soft in-game money	Miles			Hard in-game money	Loyalty points	Virtual economy money	Company-specific money	Crypto money
	US Dollars Cash money in physical or digital format that is regarded as official currency in the U.S. and is issued and controlled by the Federal Reserve. It can be converted into other official currencies (e.g., ₤, ₹) and back into US $	World of Warcraft World of Warcraft is an online computer game. Players can earn “gold” by completing different quests or selling virtual goods. Gold can be exchanged among the players. The developers forbid the purchase or resale of gold for REM, but there are ways to purchase gold illegally	United MileagePlus United’s frequent flyer miles can be acquired by flying United or affiliated airlines, using a MileagePlus credit card, earning points at partner companies (e.g., Marriott), or purchasing them with REM. Miles help establish premier status and benefits. They cannot be resold and have limited transfer opportunities. But goods, services and gift cards can be bought with miles, for self or other use or donation			Fortnite The developers of Fortnite, an online game, offer players both the possibility to earn their VC “V Bucks” through different tasks in the game and to purchase them directly in their online store, though earning possibilities of hard in-game money is limited. V Bucks cannot be exchanged among players	Starbucks Rewards For every dollar spent at Starbucks, depending on payment method, customers get up to 3 stars. These stars can be redeemed for rewards (e.g., food and drinks) but they cannot be converted back into REM	Second Life Second Life is both an online game and a virtual world. Users buy “Linden Dollars” with REM to purchase virtual goods within the Second Life world, at conversion rates set by market demand. Linden Dollars can be exchanged and sold on the official platform “LindeX”	Tomorrowland Festival goers pay with “Pearls” for food, drinks and merchandize at the music festival Tomorrowland. Pearls have a fixed exchange rate (€1.6 = 1 Pearl). Unspent Pearls are automatically refunded in € to the festival goer’s card after the festival	Bitcoin Bitcoin is a virtual currency built on top of blockchain technology, regulated by a decentralized network. Bitcoins can be purchased with and converted back into REM. The conversion rate is volatile. While El Salvador has accepted bitcoin as legal tender, China has banned its use, and India is debating a ban. Its retail acceptance is growing and consumer advertising has started
Similar examples	*Euros* *Chinese Yuan* *Indian Rupee* *Japanese Yen* *Swiss Franc* *Turkish Liras* *Jordanian Dinars*	*FIFA21 (coins)* *Guild Wars (gold)* *GTA V ($)*	*Miles&More (miles)* *AAdvantage (miles)* *Delta Skymiles (miles)* *True Blue (miles)*			*Candy Crush (gold bars)* *Clash of Clans (gems)* *Brawl Kings (gems)* *Words with Friends (power-ups)*	*Mariott Bonvoy (points)* *My Best Buy (points)* *Sephora Beauty Insider (points)*	*As of now, Linden Dollars are unique with no known similar examples. However, this may change in the future with the growth of the Metaverse*	*Leopallooza (Wylde Bucks)* *Garbicz Festival (SZORRYS)* *Casino Chips (e.g., RFID Chips at Resort Word, Las Vegas)*	*Ethereum* *Ripple* *Stellar* *XRP*

^*^Legal tender refers to the legally recognized currency in a particular economy that is accepted to cover any form of monetary debt

^**^In some cases, consumers may have the possibility to resale or acquire the closed system or unidirectional VCs on unauthorized exchange platforms. For example, selling frequent flyer miles violates the terms of service of all major frequent flyer programs. However, a limited number of United miles can be transferred with a transfer fee of $7.50 per 500 miles plus a processing fee of $30 per transaction. Thus, “not possible” refers to the lack of an authorized possibility of reselling the VC to the issuing company. However, by buying gift cards with miles, the miles can be made more fungible

^***^Many VCs are tied to a specific user account and cannot be transferred to another user (for free). However, one may have the opportunity sell his/ her whole user account outside the VC system on unauthorized third-party online platforms (e.g., thepointsking.com for airline miles). While some developers allow the exchange of VCs among the players (e.g., online role playing games such as World of Warcraft) others offer no such official possibility (e.g., FIFA21 online)

## VC schemes

The European Central Bank defines VCs as “[…] electronic money issued and usually controlled by its developers, and used and accepted among the members of a specific virtual community.”^3^ VCs can be classified into (1) closed system and (2) unidirectional or (3) bidirectional flow depending on their connection to real economy money.

### Closed system

Closed system VCs cannot be bought with or resold for REM. They must be earned to buy virtual goods within the specific closed environment, and are often referred to as “soft” in-game currency. The aim is to increase consumer engagement, monetizing it through upselling and advertising. For example, World-of-Warcraft players earn “gold”. Other examples include FIFA21 (coins), Guild Wars (gold), and GTA V ($).

### Unidirectional flow

VCs in this category are issued by the issuing company with limited fungibility across customers, restricted convertibility to REM and no legal recognition. The exchange rates vary and are set by the issuing company, and the purpose is to encourage engagement and repurchase with the company and its partners.

Consumers can acquire unidirectional VCs by buying them *directly* with REM or *indirectly* by spending REM. This leads to three types of VCs where the acquisition is through:

- Indirect and direct purchase: Consumers can trade in VCs earned through flights, hotel stays, or credit-card usage, for products and services from the issuing company (e.g., Marriott-Bonvoy), or products available in their marketplace (e.g., United MileagePlus). There is limited transferability across users although VCs can be monetized by converting them into gift cards/products to be gifted, used, or donated.
- Direct purchase and engagement: Referred to as “hard” in-game currency, such as CandyCrush (gold bars), Clash of Clans (gems), Brawl Kings (gems), and Words-with-Friends (power-ups).
- Indirect purchase: Typical of loyalty programs, VC points are used to reward loyal customers and encourage repurchase (e.g., Starbucks stars, Best Buy and Expedia points, and Macy’s Money).

Unidirectional VCs cannot be converted back to REM without resorting to unauthorized third-party platforms.

### Bidirectional flow

Bidirectional flow VCs share many similarities to REM. They can be exchanged from and into REM, but they lack legal recognition and are not issued by a central bank. There are three types: virtual economic money (e.g., Second Life’s tradable Linden dollars), company-specific money (e.g., Tomorrowland Pearls, virtual casino chips), and crypto currencies (e.g., bitcoin). Companies issue company-specific VCs for payments within their ecosystem. For example, Tomorrowland’s “Pearls” must be purchased with REM, used for all payments at the festival, with unused Pearls refunded.

## Research opportunities

VCs open new avenues for academic research. VC features (Table 1) are antecedents to seven information processing stages discussed below, with user characteristics moderating these relationships. Consequences include spending and post-transaction outcomes. Research questions that arise from this framework are presented below.

### Economic utility

Economic utility refers to a VCs purchase price and its expected appreciation/ depreciation, volatility, and REM convertibility. Companies choose their own conversion rate when introducing a VC which can be either a fraction or a multiple of REM (e.g., Pearls = €1.6; $1 = 100 miles = 0.000015 BTC), a factor that can affect spending (Raghubir & Srivastava, 2002).

- *Do VC exchange rates influence consumers’ price perceptions and purchase behavior?*
- *Is price elasticity contingent on VC versus REM?*

Exchange rates of VCs can be pegged to REM (e.g., Pearls-€), be determined by market supply and demand (e.g., Bitcoins), or by company-related factors (e.g., miles devaluation).

- *How do different exchange rate schemes, and their stability, influence their perceived value and affect purchase behavior, satisfaction and loyalty?*
- *How do usage and purchase behavior change when the VC is considered a stored value versus a medium of exchange?*
- *Does devaluation affect spending of VCs, loyalty, satisfaction, and engagement?*
- *Do fluctuating and volatile exchange rates increase price complexity?*
- *Can increasing VC fungibility across customers increase engagement?*

Many hard in-game currencies can be bought in different bundles with volume discounts (e.g., 6 packages of “CandyCrush gold bars”, with the price/bar half in a 1000- vs. 10-bar pack), and be used to purchase additional in-game packages, leading to several different prices.

- *How do consumers account for different exchange rates?*
- *Do consumers’ expected volume discounts differ for REM versus VC?*
- *Does price complexity affect purchase intentions, satisfaction, and engagement?*

### Transaction utility

Transaction utility relates to the acceptance of a VC. Paying with company-specific (vs. fungible) funds increases consumer preference for company-typical products (Reinholtz et al., 2015).

- *Are VCs typically used for purchases similar to how they were earned?*
- *What individual differences moderate the likelihood of saving versus spending a VC?*

Priornted that consumers spend more using credit cards and gift certificates than they do using cash (Raghubir & Srivastava, 2008). This suggests:

- *Are company-specific VCs more likely to be spent versus REM?*

### Accounting

Accounting refers to the mental accounts that consumers use to categorize their VCs (with each account used for a different purpose), and includes whether a VC is thought of as a resource to be spent (vs. invested in). On occasion, VCs are issued into two separate accounts (e.g., United’s Miles, PlusPoints).

- *Do consumers have different mental accounts for different VCs from the same issuing company?*
- *Do consumer include VC balances in their net worth assessment?*

### Perceptions

Perceptions include the perceptual fluency of a VC as well as consumers’ price perceptions. When paying with gift cards, consumers try to avoid unspent funds (White, 2008), sometimes leading them to spend over the gift card’s face value. Uneven prices in in-app purchases lead to residual values.

- *Do residual values in VCs enhance spending or purchase avoidance?*
- *Do residual values affect post-purchase satisfaction and engagement?*

### Cognitions

Cognition includes the similarity of a VC to REM, price-complexity, conversion-ease, and perceived value. The lower the similarity, the lower the negative feelings associated with spending, and the higher purchase likelihood, especially for hedonic products (Thomas et al., 2011).

- *How similar do consumers perceive different VCs to be to cash?*
- *What are the antecedents of these similarity perceptions?*
- *What offerings should loyalty programs offer to encourage purchase?*
- *What individual differences moderate the effect of perceived similarity on spending?*

Consumers depreciate costs over time (Gourville & Soman, 1998), with longer gaps making costs less relevant at the time of consumption.

- *Do consumers depreciate VC costs over time?*

### Affect

Pain-of-paying is a negative affective reaction, and positive feelings associated with higher loyalty status are positive affective reactions. Shah et al. (2016) showed that the greater the pain-of-payment, the greater brand attachment.

- *Is pain of payment lower for VCs than for REM?*
- *Is brand attachment lower when purchased with VCs?*

### Memory

Memory refers to the strength of the memory trace for the price paid, and is weaker for credit cards versus cash (Srivastava & Raghubir, 2002).

- *Is the memory trace for a VC weaker than REM?*
- *Does a weaker memory trace encourage VC spending?*

### User characteristics

Approximately 3.5% of gamers spend REM on hard in-app money.^4^

- *What personality traits increase likelihood to spend REM on VCs, and VCs?*
- *What policies should be implemented to prevent overspending?*

Alter and Oppenheimer (2008) show that consumers who pay with unfamiliar (vs. familiar) forms of currency believe they have less purchasing power.

- *Does a VCs name affect familiarity perceptions?*
- *How does consumer familiarity with a VC affect processing fluency of VC prices and purchase intentions?*

## Conclusion

VCs are a prevalent, growing, yet understudied, subject in marketing. Their shared features and their heterogeneity offer novel research questions along the purchase process, especially as virtual environments bear untapped potential to predict real-world behavior (e.g., Lofgren & Fefferman, 2007).

## Data Availability

Not applicable.

## Footnotes

1 Mordor Intelligence (2022). Global Gaming Market—Growth, Trends, COVID-19 Impact, and Forecasts (2022—2027). Retrieved February 11, 2022, from https://www.mordorintelligence.com/industry-reports/global-gaming-market

2 Daily Beast (2022). Matt Damon’s Viral Crypto Ad Is an Embarrassing Cash Grab. Retrieved February 11, 2022, from https://www.thedailybeast.com/matt-damons-viral-crypto-ad-is-an-embarrassing-cash-grab

3 European Central Bank (2012). Virtual Currency Schemes. Retrieved February 11, 2022 from http://www.ecb.europa.eu/pub/pdf/other/virtualcurrencyschemes201210en.pdf

4 AppsFlyer (2016). The State of In-App Spending: Golbal & Regional Benchmarks. Retrieved February 11, 2022 from https://cdn2.hubspot.net/hubfs/597489/IAP_Guide/The_State_of_In-App_Spending_AppsFlyer.pdf

## References

[CR1] Alter AL, Oppenheimer DM (2008). Easy on the mind, easy on the wallet: The roles of familiarity and processing fluency in valuation judgments. Psychonomic Bulletin and Review.

[CR2] Gourville JT, Soman D (1998). Payment depreciation: The behavioral effects of temporally separating payments from consumption. Journal of Consumer Research.

[CR3] Lofgren, E. T., & Fefferman, N. H. (2007). The untapped potential of virtual game worlds to shed light on real world epidemics. *The Lancet,**7*(September), 625–629. 10.1016/S1473-3099(07)70212-8 10.1016/S1473-3099(07)70212-8 17714675

[CR4] Raghubir, P., & Srivastava, J. (2002). Effect of face value on product valuation in foreign currencies. *Journal of Consumer Research,**29*(3), 335–347. 10.1086/344430

[CR5] Raghubir, P., & Srivastava, J. (2008). Monopoly money: The effect of payment coupling and form on spending behavior. *Journal of Experimental Psychology: Applied, 14*(3), 213–225. https://doi.apa.org/doi/10.1037/1076-898X.14.3.213 10.1037/1076-898X.14.3.213 18808275

[CR6] Reinholtz N, Bartels DM, Parker JR (2015). On the mental accounting of restricted-use funds: How gift cards change what people purchase. Journal of Consumer Research.

[CR7] Shah AM, Eisenkraft N, Bettman JR, Chartrand TL (2016). “Paper or plastic?”: How we pay influences post-transaction connection. Journal of Consumer Research.

[CR8] Srivastava, J., & Raghubir, P. (2002). Debiasing using decomposition: The case of memory-based credit card expense estimates. *Journal of Consumer Psychology,**12*(3), 253–264. 10.1207/S15327663JCP1203_07

[CR9] Thomas M, Desai KK, Seenivasan S (2011). How credit card payments increase unhealthy food purchases: Visceral regulation of vices. Journal of Consumer Research.

[CR10] White, R. (2008). The mental accounting of gift card versus cash gift funds. *Advances in Consumer Research*, *35*, 722–723.

